# Exploration on the collaborative innovation path of college students' ideological education and psychological education

**DOI:** 10.3389/fpsyg.2022.969508

**Published:** 2022-08-26

**Authors:** Cuixia Lin, Keneng Lin

**Affiliations:** ^1^Moral Culture Research Center, Hunan Normal University, Changsha, China; ^2^School of Foreign Language, Hulunbeier University, Hulunbeier, China

**Keywords:** college students, ideological and political education, psychological health early warning, psychological education, structural equation modeling

## Abstract

As a highly practical educational activity, mental health education must be guided by rich theories to maintain the steady development of mental health education. Ideological education plays a predictive role in college students' crisis management, which can improve college students' psychological crisis management ability. This paper proposes an early warning index system and applies it to the construction of an early warning mechanism, completes the qualitative and quantitative analysis of early warning information evaluation, and changes the situation that information evaluation only stays at the level of qualitative analysis in traditional methods. Quantitative analysis is more conducive to accurately predict the occurrence of psychological crisis. Through empirical research, this paper finds that there is a significant interaction between stressors and coping styles in the process of affecting mental health. The result of interaction is not only equal to the superposition of the influence of a single factor, but also greater or lesser than the superposition of the influence of a single factor. The study found that there was a significant correlation between stressors and mental health. It is one of the many factors that affect mental health, and it is also the main reason to induce college students' psychological crisis. Mature coping styles are significantly positively correlated with mental health, while immature coping styles are significantly negatively correlated with mental health. This paper tests students, which is more conducive to the timely warning of psychological crisis.

## Introduction

Ideological education and psychological health education complement and promote each other. They can also learn from each other and complement each other, so as to jointly play the positive role of educating, cultivating, and shaping a harmonious personality of all-round development. Psychological health education, as a highly practical educational activity, must be guided by rich theories to maintain the steady pace of development of psychological health education. If there is no rich theory to support, it will inevitably affect the development of psychological health education practice (Lipson et al., 2019). Psychological health education is an important part of ideological education in colleges and universities. The theoretical research on psychological health education also enriches the content of ideological education and improves the pertinence of ideological education. To predict what kind of thinking a person will have in a certain environment, it is necessary to have a comprehensive understanding of his psychology (Son et al., 2020). An ideological and political worker should grasp the ideological and psychological characteristics of students by grade. At the same time, workers should also study the characteristics of different groups of students at the same school level, establish a personalized educational concept, comprehensively grasp the law of ideological generation, and change, to grasp the personality of the work object, so as to accurately predict a person's thoughts, to detect and identify potential and actual crises in time, to take measures to reduce the mutation and unexpectedness of crises, and to prevent the occurrence of psychological crises. Therefore, psychology is able to predict the predictability of ideological and political work.

Ideological and political education refers to the use of certain ideological concepts, political views, and moral norms by society or social groups, which exert purposeful, planned, and organized influence on its members, so that they can form social practice activities that meet the ideological and political morality required by a certain society. Ideological and political education is an important part of education in our country. With the development of culture and concept diversification, its position in higher education in our country is becoming more and more prominent. Generally speaking, college students' ideological and political education has four obvious characteristics: political, practical, contemporary, and penetrating. College students' mental health education is based on the physiological and psychological development characteristics of college students, using relevant psychological education methods and means. It is an important part of quality education to cultivate college students' good psychological quality, promote the comprehensive and harmonious development of college students' body and mind, and improve their quality in an all-round way. It is an important link to implement the cross-century quality education project and cultivate cross-century high-quality talents. Ideological education and psychological health education are mutual conditions and prerequisites. A healthy psychology makes it easier for students to accept ideological education and internalize their beliefs and externalize their behaviors (Lee and Jung, 2018). Psychological health education can provide psychological conditions for the effective implementation of ideological education, and it is also a reasonable extension and extension of the goals and contents of ideological education in colleges and universities and also provides methods for ideological education. It is an important responsibility of ideological and political workers to think about certain contents of political education, such as situation policy education and line policy education, but psychological health educators rarely get involved. Ideological and political educators can use the method of psychological counseling in psychological health education to help, inspire, and educate the subjects, remove the psychological barriers in the formation and development of their thoughts, help them change the angle of looking at problems, adjust the methods of looking at problems, and establish a new way of thinking and establishing a positive and enterprising spirit (Dunbar et al., 2018). For problems such as various neuroses, interpersonal disorders, personality disorders, and emotional disorders, although ideological and political workers subjectively want to solve them, they are often overwhelmed and lacking in effort. For solving such problems, psychological health educators are often able to do it well, and they can fill in the dead corners that ideological education cannot take care of.

The occurrence of college students' psychological crisis has profound internal reasons. Its occurrence is not isolated or accidental. It is usually affected by some special events, and there are certain signs before it occurs (Liu et al., 2019). Therefore, the psychological crisis of college students can be forewarned. As long as possible crisis factors are found early, these factors are reasonably evaluated, and effective intervention and preventive measures are taken, the occurrence of crises can be avoided. Stressors are part of the mediation between social support and stress response. On the one hand, social support has a direct impact on stress response. The influences are all significant. Based on the interaction effect of latent variables, this paper proposes a psychological crisis early warning index system for college students and establishes a structural equation model with interactive terms to explore the path relationship between factors that induce psychological crisis and psychological crisis. The interaction effect was analyzed (Hu and Li, 2018). Based on the score of psychological health, it can quickly evaluate the degree of the crisis state of the students. According to the results of the early warning evaluation, different intervention measures are taken according to the degree and state of the psychological crisis of the students. Coping styles play an important role in regulating the relationship between stressors and psychological health. Faced with the same degree of stress, different coping styles will bring different stress feelings to individuals. Therefore, psychological health educators should pay more attention to the cultivation of mature coping styles of college students and try to avoid the harm of immature coping styles to college students' psychological health.

The innovation point of this paper: The fundamental purpose of college students' psychological health education is to cultivate and improve the psychological quality of college students, optimize the psychological quality of students, and improve their personality. The ideological and political work of colleges and universities attaches great importance to cultivating the ideological and moral quality, scientific and cultural quality, ability quality, and physical quality of college students. The predictive role of ideological education in crisis management of college students is to collect relevant information about crisis events through ideological and political work before the occurrence of crisis events, to reasonably predict the ideological conditions, social psychology, and their negative effects that will occur, to strengthen crisis awareness education, to improve the crisis management ability, to formulate scientific preventive measures, and to lay a solid foundation for the correct response to the crisis. This paper proposes an early warning index system and applies it to the construction of an early warning mechanism to complete the qualitative and quantitative analysis of early warning information evaluation, which changes the situation that the information evaluation in traditional methods only stays at the level of qualitative analysis. Quantitative analysis is more conducive to accurately predict the occurrence of psychological crisis.

This paper tests students, which is conducive to the timely warning of psychological crisis. The research is divided into five parts. The first part introduces the research and development background of relevant scholars on college students' mental health and ideological education. The second part analyzes the connotation of college students' mental health education. The third part establishes structural equation model indicators according to the mental health status of college students. This paper analyzes the influencing factors of college students' psychological crisis prediction. The fitting test of the model is carried out. The fourth chapter analyzes the role of ideological education in psychological crisis prediction. Then, the exploratory statistical data and impedance factor analysis are carried out. Finally, the full text is summarized. Through empirical research, this paper finds that there is a significant interaction between stressors and coping styles in the process of affecting mental health. There is a significant correlation between stressors and mental health. It is one of the many factors that affect mental health, and it is also the main reason to induce college students' psychological crisis.

## Related work

Ideological education dissemination of culture is not the original copy of the original culture, but a process of selection. It is imperative for colleges and universities to strengthen cultural choices in ideological education. The construction of campus culture must keep pace with the times, be pioneering and innovative, and make the socialist progressive culture the direction that leads the cultural construction of college students, help college students establish a correct cultural outlook, and improve cultural awareness. The university stage is a lifelong growth of a person. The important stage and golden period are also the key stages for the formation of outlook on life, world outlook, and values. At this stage, the psychological development of college students tends to mature, but it has not reached the real stability. To understand the connotation of college students' psychological health education, we must first understand the meaning of psychological health, what impact and role it has on people's physical and mental development, and what the specific standards of psychological health are. Only by clarifying these issues can we talk about college students and the concept of psychological health education (Guo et al., 2022).

Hahn et al. built a psychological crisis early warning system for college students and proposed to strengthen the prevention and management of college students' psychological crisis, establish an early warning mechanism composed of society, schools, and families, and build various types of crisis management systems (Hahn et al., 2020). Jin et al. believe that crisis is a temporary psychological imbalance caused by sudden major life events. It is a double-edged sword, which can cause anxiety, sadness, resentment, and other negative emotions and can make people more mature. Normal people are in a state of physical and mental balance. In their daily life, their thinking, will, emotion, and physiological needs are in a state of harmony to a certain extent. When uncomfortable stress occurs, people's balance state can be affected, and emotions and thinking may appear, which is out of control, so as to experience an extreme emotional disturbance, when people are in crisis (Jin et al., 2020). Alonso et al. believe that a crisis is a period of psychological imbalance, when an individual encounters critical consequences caused by major problems, or is in a crisis situation, and cannot use previous coping strategies to deal with this situation (Alonso et al., 2018). Sontag et al. believe that crisis vulnerability and cognitive factors are important influencing factors of college students' handling of crisis events, so he has a certain theoretical conception of college students' crisis handling methods (Sontag-Padilla et al., 2018). Lin et al.'s study found that people with active personality characteristics are not easily controlled by the situation; therefore, they can cope with stress more actively, showing flexibility and initiative in coping (Lin et al., 2020). Chang et al. think that ideological education and psychological health education are related and different at the same time, and they complement each other. Ideological education includes psychological health education in extension, and psychological health education is an important content of ideological education. Both are important guarantees for the healthy growth and success of college students (Chang et al., 2020). Ibrahim et al. found that with age, the frequency of using mature coping styles increased, while the frequency of using immature coping styles decreased (Ibrahim et al., 2019). Kecojevic et al. believe that stable factors include individual age, gender, genetic quality, and personality traits. The study believes that the influence of personality factors on coping style is restricted by situational variables, and personality factors may affect the nature and type of coping style. Situational factors, namely unstable factors, mainly include the objective characteristics of the stressful situation and the individual's subjective understanding and evaluation of the situation (Kecojevic et al., 2020). Qi and Yao believe that in addition to personal factors, there are social and situational factors that affect coping styles. We believe that when the stressor of the objective environment interacts with the individual, the objective environment does not change according to the subjective factors of the individual, and the individual does not change according to the nature, type, and intensity of the objective stressor. Therefore, the objective environment and individual factors can be classified as stable factors. When the stressor interacts with the individual, it constitutes a situation, and the social support at this time becomes the big situation in the situation, so the individual's evaluation of social support can also be regarded as a situational factor (Qi and Yao, 2021). Gopalan et al. believe that college students' psychological crisis early warning means that colleges and universities fully mobilize all available resources, actively take various possible and feasible measures, strive to prevent and limit the occurrence of various crisis behaviors, help existing crises as much as possible, and resolve and minimize the losses caused by the crisis. The purpose is to effectively provide accurate information for psychological crisis intervention and ultimately improve students' psychological quality to help them grow (Gopalan et al., 2022).

In general, the psychological health education of college students can improve the pertinence of ideological education. Under the condition of immature ideological quality and psychological quality, college students are vulnerable to the environment of many negative factors. College students' psychological health education is the premise and foundation for the smooth development of ideological education. The fundamental tasks of the two are consistent with the object of education. They both take college students as the object of education and use different methods and means to guide college students to improve their ideological and moral cultivation, enhance their political awareness, and cultivate students with positive personality quality and health. In-depth study of the new educational route of the integration of college students' psychological health education and ideological education will effectively promote the smooth development of ideological education in colleges and universities. Although the occurrence of college students' psychological crisis is often sudden, we do not know when it will happen and what form it will take, but this does not mean that we are helpless. As long as the abnormal indicator information is reasonably analyzed and scientifically evaluated, the possibility of a crisis can be more accurately predicted and prepared for.

## Analysis of the interaction effect of latent variables based on the psychological health of college students

### Structural equation model of college students' psychological health

With the development of social sciences, psychology, education and economics, and other disciplines, higher requirements are put forward for in-depth research on hidden variables. Therefore, the research on structural equation models that can handle hidden variables well is also increasing deeper. Structural equation modeling is widely used in educational psychology and has become an important data analysis method in this field (Savage et al., 2020). Structural equation modeling is a statistical method that uses the covariance matrix of variables to analyze the relationship between variables. The starting point is to establish a causal hypothesis relationship model between observed variables.

**Figure 1:**
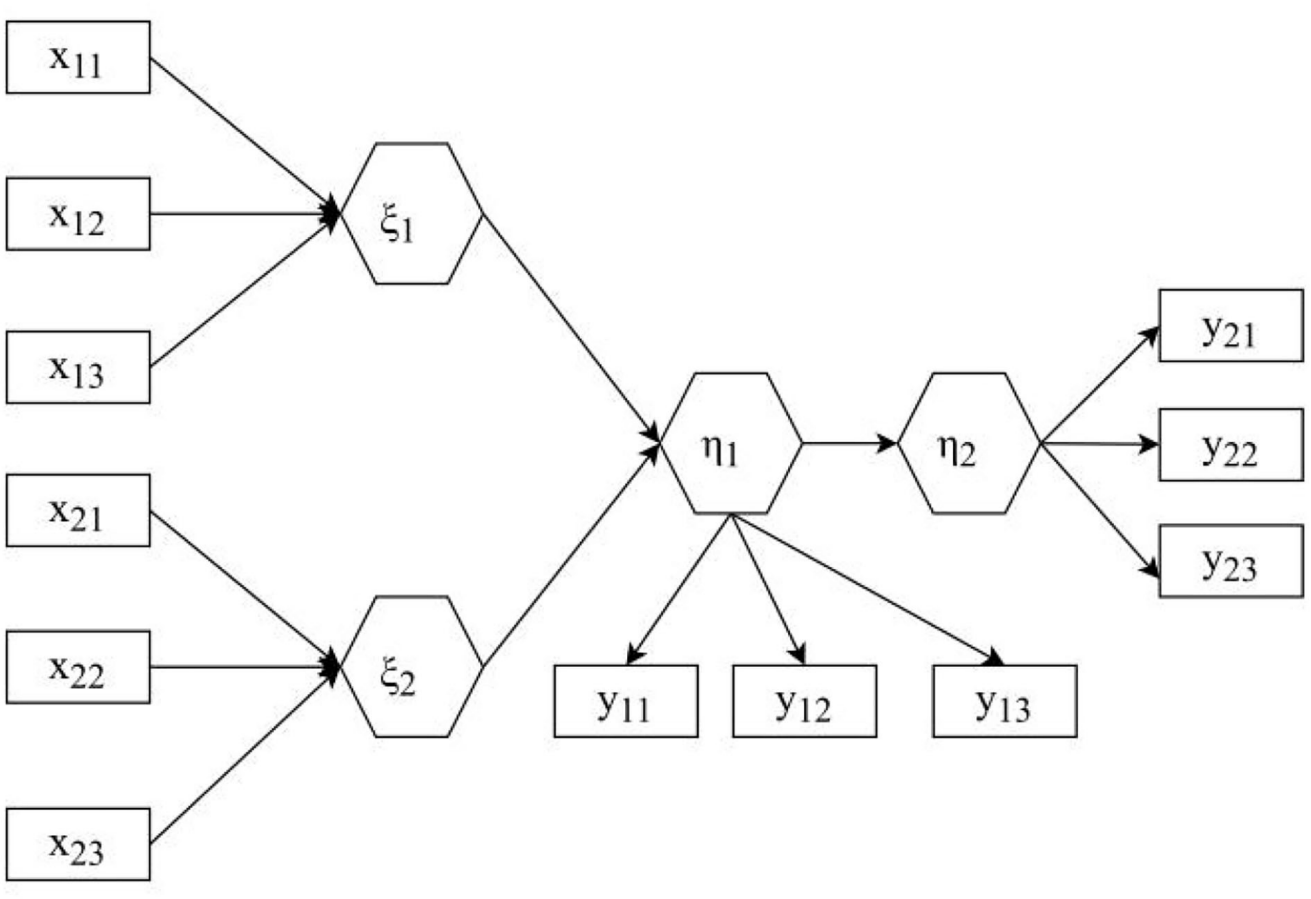
Structural equation model.

Observed variables and latent variables that can be directly observed and measured are called observed variables and can also be called explicit variables or indicator variables, such as students' test scores and parents' income. Variables that cannot be obtained by direct observation or measurement are called latent variables, which can also be called latent variables or latent variables, such as academic achievement, socioeconomic status of the family, and scientific ability (Hirsch et al., 2019). We need to find suitable observed variables to describe latent variables accurately and reasonably and, at the same time, establish a certain constraint relationship between observed variables and latent variables to analyze and evaluate latent variables. Before establishing a structural equation model, it is necessary to clarify the hidden variables and explicit variables that need to be considered in the model, to determine the relationship between hidden variables, explicit variables, and hidden variables, and to determine the basic structure of the structural equation model. Dealing with the relationship between multiple results, or encountering variables that cannot be observed directly, these are the problems that traditional statistical methods cannot solve well. Traditional statistical modeling and analysis methods cannot effectively deal with latent variables, while structural equation models can deal with latent and explicit variables at the same time. Traditional linear regression analysis does not allow multiple dependent variables to have measurement errors, and it is assumed that the independent variables have no errors. Structural equation models do not have these limitations. That is to say, it is necessary to establish the measurement equation and the structural equation. The structural equation model consists of two parts, namely the measurement equation and the result equation (Tucker, 2021). The structural equation model is shown in Figure 1.

The relationship between hidden variables is shown in formula (1).


(1) $$\begin{array}{r} \eta =B\eta +x\xi +\xi \end{array}$$

Among them, η represents the vector composed of endogenous explicit variables, *B* represents the relationship between endogenous latent variables, and ξ represents the vector composed of the residuals of the structural equation, reflecting the unexplained part of the structural equation.

The covariance matrix of exogenous explicit variables is shown in formula (2).


(2) $$\begin{array}{r} \sum x\left(\theta \right)=B\eta \Phi +x\xi \end{array}$$

Modeling parameters can be applied to structural equation models. The model evaluation of structural equation models is divided into two types: partial evaluation and overall evaluation. The goodness of fit of the model is evaluated. Part of the evaluation is to evaluate the individual equations, parameters, and variables in the structural equation model alone (Wang, 2019).

The invertible variance matrix is shown in formula (3).


(3) $$\begin{array}{r} \sum y\left(\theta \right)=B\eta^{\prime }\Phi -x\xi \end{array}$$

The covariance matrix of *x* and *y* is shown in Equation (4).


(4) $$\begin{array}{r} \sum xy\left(\theta \right)=\eta_{y}B\delta x\xi \end{array}$$

In the process of model evaluation, if it is found that the goodness of fit of the established structural equation model is not good enough, it is necessary to revise the model. The revision of the model includes two parts, one is the adjustment of the measurement model, and the other is the adjustment of the structural model. The covariance matrix of exogenous explicit variables and endogenous explicit variables is shown in formula (5).


(5) $$\begin{array}{r} \sum xy\left(\theta \right)=EBx\Phi_{y}A_{x} \end{array}$$

Assuming that the matrix is invertible, shift η is shown in (6).


(6) $$\begin{array}{r} \eta ={\left(B-I\right)}^{2}\left(x\xi -\xi^{-1}\right) \end{array}$$

The adjustment of the measurement model is mainly to adjust the relationship between the explicit variable and the hidden variable, that is, to increase or decrease the number of explicit variables corresponding to a hidden variable making adjustments. The adjustment of the structural model is mainly the adjustment of the correlation between the hidden variables or the increase or decrease of the quantity. The measurement equations of the indicators are shown in (7) and (8).


(7) $$\begin{array}{r} y_{B}=\eta_{1}+\varepsilon_{y} \end{array}$$

(8) $$\begin{array}{r} y_{x}=\lambda_{x2}\eta +\varepsilon_{x} \end{array}$$

Traditional factor analysis has many limitations. For example, an indicator or topic can only be subordinated to a single factor and cannot be subordinated to multiple factors, and high-order factors are not considered. The model processed by structural equation is relatively complex, and it can deal with one index subordinate to multiple factors or consider the high-order factor model with a complicated subordination relationship.

### Evaluation of structural equation models

Model evaluation is to judge whether the set model matches the collected data and the degree of matching from the structural equation model as a whole. That is, the variables in the model are evaluated individually. In the practical application of structural equation modeling, the identification of the model is actually difficult to judge. The degree of freedom can be calculated. If the degree of freedom is ≥0, the model is nothing else. For general models, a sufficient and necessary condition has not been found that can be used to judge whether the model is identifiable (Ebert et al., 2019). The definition of the covariance matrix between variables when the model is established is shown in (9).


(9) $$\begin{array}{r} RM=\sqrt{\max\left(\frac{1}{N}-\frac{f_{ML}}{df}\right)} \end{array}$$

A result index of the above formula below 0.15 indicates a good fit, and below 0.06 indicates a good fit. The structural equation is regarded as a causal model between observables, that is, the endogenous latent variables and exogenous latent variables are regarded as observable variables and are judged according to the identification method of the causal model. Conditions are shown in Table 1.

**Table 1:** Causal model conditions.

**Identification rule**	**Identify objects**	**Condition requirements**	**Sufficient and necessary conditions**
T-law	Model	T is <2	Sufficient condition
Recursion	Model	A triangular matrix is a diagonal matrix	Sufficient condition
Zero b	Model	0	Necessary conditions
Order condition	Equation	Variables in the equation are at least 1	Necessary and sufficient condition
Rank condition	Equation	It is 1 in the equation variables, and the rest of the variables are freely estimated	Necessary conditions

The method of estimating the unknown parameters in the model is according to the measured sample data, and the maximum likelihood estimation is to make the fitting function as shown in formula (10).


(10) $$\begin{array}{r} F_{UIS}\frac{1}{2}tr\left[\left(\sum {\left(s\right)}^{2}-S\right)\right] \end{array}$$

After parameter estimation, the parameter fitting model can be obtained, but to know the quality of the fitting model, it is necessary to evaluate the model. If a new latent variable is added to the model in the study, if traditional statistical methods are used, the newly added variable does not affect the correlation between other variables, and the calculation result is exactly the same as the calculation result of the original model, indicating that the addition of a new variable does not affect the relationship between other variables, but in the structural equation analysis, the addition of a new variable will affect the relationship between other variables, indicating that the addition of a new variable affects the internal structure of the variable (Wu et al., 2020). The method of identifying all variables as exogenous variables is identified using the model identification method of the confirmatory factor model. The model identification method of the confirmatory factor model is shown in Table 2.

**Table 2:** Confirmatory factor model.

**Identification rule**	**Condition**	**Sufficient and necessary conditions**
The rule of three indicators	At least 3 indicators per factor, each indicator measures only one factor errors are not correlated	Necessary conditions
Two-index rule	Each indicator measures only 1 factor	Necessary and sufficient condition
T-law	Each factor has a factor associated with it	Sufficient condition
Law of designation	T is not less than 0.5	Necessary and sufficient condition

In structural equation model analysis, multiple dependent variables are considered and processed at the same time. In traditional statistical methods, even if multiple dependent variables are given in the result chart after statistical analysis, the calculation in traditional statistical methods. The process calculates each variable independently and ignores the existence of other variables when calculating the influencing factors of a certain variable. Hidden variables such as psychological pressure and psychological tolerance cannot simply be measured by a single indicator, so generally speaking, the structural equation model analysis with errors allows both dependent and independent variables to contain measurement errors and use structures. The correlation coefficient or regression coefficient between the hidden variables calculated by the equation analysis may be very different, and the magnitude of the difference is mainly related to the factor loading of the explicit variables on the hidden variables (Oswalt et al., 2020).

## Design of psychological crisis early warning system for college students

### The role of ideological education in psychological crisis prediction

Crisis events of college students are different from ordinary social crisis events. The main group of crisis events are educated college students (Elmer et al., 2020). They have their own ideas and enthusiasm for life, and they are also very concerned about the development of society. However, the fragile psychology of college students can easily lead to thinking and psychological distortions (Zdravković et al., 2022). College students are also easy to be used by people with ulterior motives in the society. Because the Internet is developing rapidly and technology is advanced, it is easy for bad guys to disguise themselves to deceive college students and lead them astray. Once an incident occurs, it must be dealt with in time; otherwise, it will increase the harm. There will be enormous pressure, and events will form a chain reaction. The predictive role of ideological education in crisis management of college students is to collect relevant information about crisis events through ideological and political work before the occurrence of crisis events, to reasonably predict the ideological conditions, social psychology, and their negative effects that will occur, to strengthen crisis awareness education (Patsali et al., 2020), to improve the crisis management ability, to formulate scientific preventive measures, and to lay a solid foundation for the correct response to the crisis.

Crisis prevention is more important than crisis management. The best state of crisis management is to eliminate the crisis in the bud and avoid it from happening (Gupta et al., 2018). Ideological education plays a guiding role in intervening in the rational crisis of college students. The guiding role of ideological education is mainly reflected in the orientation of ideology and behavior. Strengthening crisis prevention is the premise and guarantee of college students' crisis management. We have adopted strict system and careful inspection to foresee the crisis that college students may face. Under normal circumstances, there will be some signs before the crisis. University administrators can find potential signs of crisis by paying close attention. Only by paying attention to crisis early warning and establishing a good crisis early warning system can we effectively avoid or slow down the occurrence of many college students' crisis events. The ideological orientation of ideological education is mainly to cultivate college students' sense of crisis, adjust students' view of crisis, and eliminate students' panic psychology. Therefore, the occurrence of many college students' crisis is closely related to the lack of crisis early warning system (Castaldelli-Maia et al., 2019; Harrer et al., 2019). In terms of ideological orientation, due to the sudden and dangerous nature of the placement and management crisis of college students, people often feel panic, resulting in emotional fluctuations and misunderstanding of crisis events. The crisis of most college students is predictable. Only by timely prediction can the crisis be prevented before it occurs. Therefore, it is very important to pay attention to the crisis early warning function. Only by paying attention to crisis early warning and establishing a good crisis early warning system can we effectively avoid or slow down the occurrence of many college students' crisis events.

### Data investigation and factor analysis

To further understand the mental health problems of college students and give them mental health counseling earlier, the study investigated and analyzed the mental health problems of college students. This activity takes the students of Humanities College as the sample and carries out sampling survey in the form of questionnaire survey. The purpose of the survey was to investigate the mental health of college students. This paper conducts a random questionnaire survey on a university student population, classifies the factors that affect stressors, determines the dimensions of stressors included in college students, and selects all levels of indicators of stressors, laying a foundation for the construction of psychological crisis early warning indicators for college students, that is data theory foundation.

The purpose of factor analysis is to find out the underlying structure of the questionnaire, reduce the number of item items, and turn it into a set of simplified but highly correlated variables, so as to test whether the questionnaire can effectively measure the characteristics that are supposed to be measured. Seven common factors were extracted by principal component analysis, and the factor loading matrix results after data processing are shown in Table 3.

**Table 3:** Factor loading matrix.

**Stressor**	**Item**	**Load matrix**
Front pressure	7~12	0.617
Study-induced stress	1~6	0.767
Independence and independence pressure	21~29	0.776
Social and interpersonal stress	13~18	0.788
Heterosexual relationship pressure	30~35	0.725
Family and financial stress	19~20	0.512
Major sudden stress	36~39	0.689

The relevant statistical analysis was carried out on the survey data, and the mean and standard deviation were used to analyze the overall characteristics of stressors, coping styles, and psychological health of college students.

**Figure 2:**
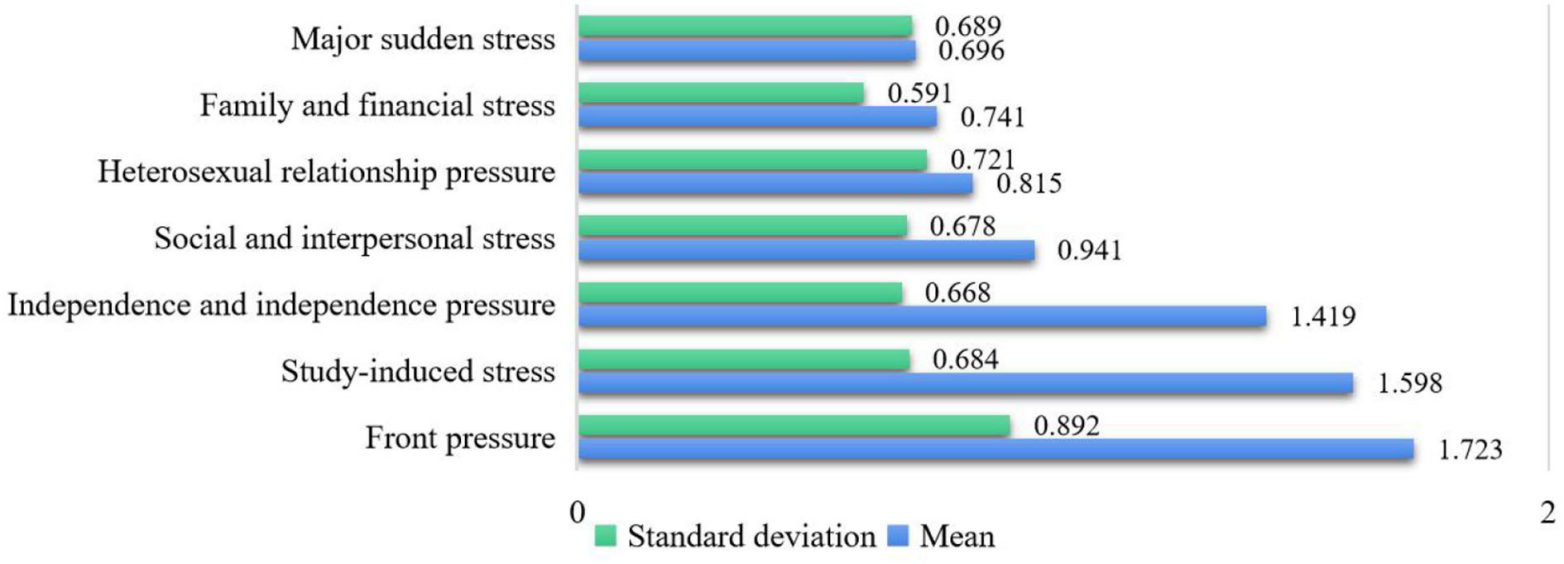
Score statistics of each dimension of college students' stressors.

**Figure 3:**
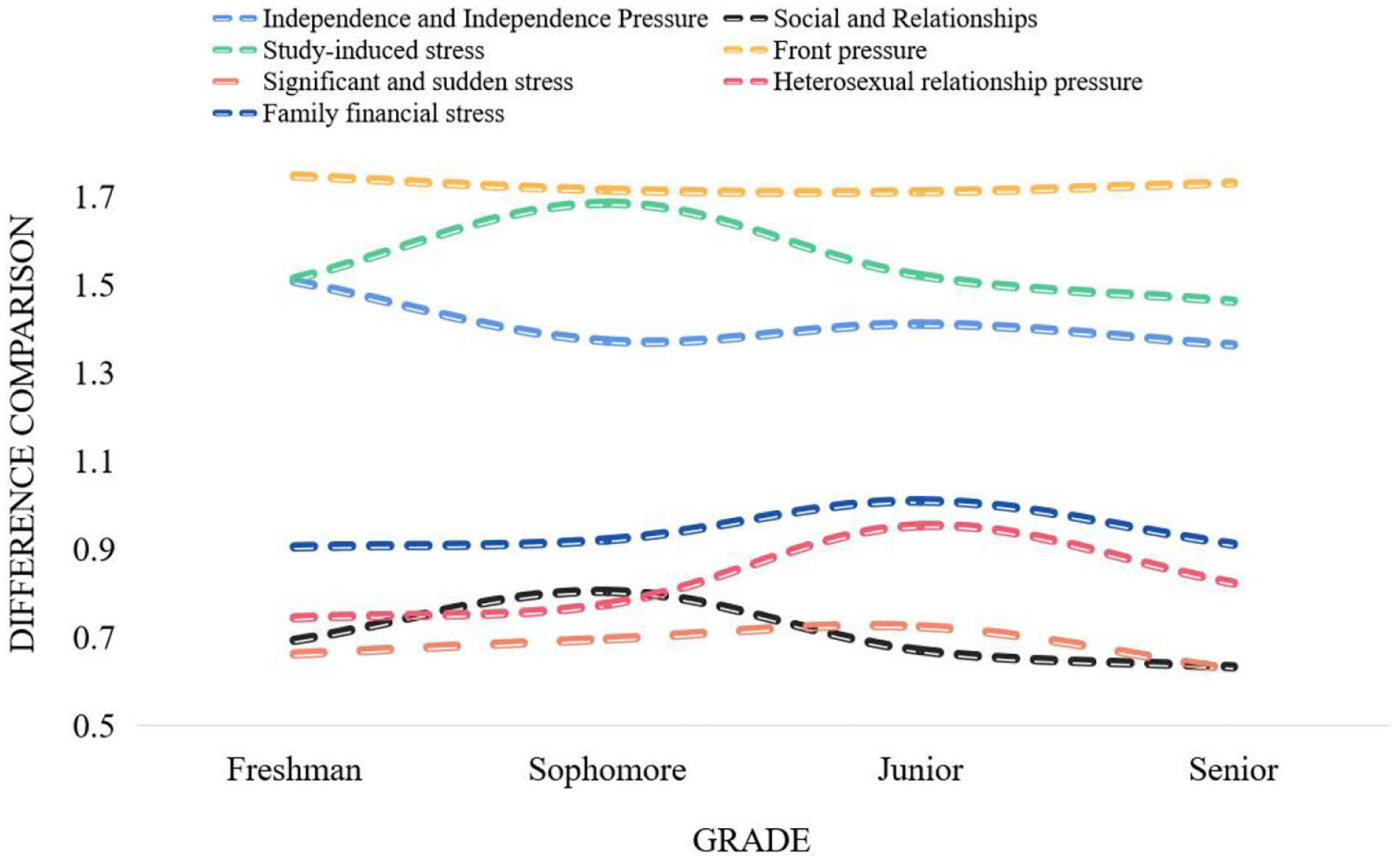
Different stressors of college students in grades.

As can be seen from Figure 2, the influence of stressors on students' psychological pressure is from strong to weak, and the order is the pressure of the future, the pressure of learning, the pressure of autonomy and independence, the pressure of family and economy, the pressure of heterosexual relationships, and the pressure of social and interpersonal relationships and significant and sudden stress, which explain that the future and learning are the two things that make students feel the most pressure. The differences in grades of stressors among college students are shown in Figure 3.

One-way analysis of variance was performed on the scores of each dimension of stressors of college students of different grades. Freshmen are the most stressed in terms of their future and autonomy and independence. This is because with the expansion of college enrollment and the saturation of the job market, college students have indicators of the difficulty of finding employment before they enroll. Freshmen are thinking about finding a job after graduation, making plans for their future, and troubled by employment, I dare not relax for a moment. Sophomores are more stressed in their studies and social and interpersonal relationships. This is because the sophomore year is regarded as the golden stage of four-year university study. At this time, there are not only many types of professional courses, but also more difficulty, and various grade examinations are also coming, so students are under great pressure to study. What troubles juniors and seniors the most is family and financial pressure and heterosexual relationship pressure. The older the students, the deeper the understanding of poverty, and the greater the economic pressure on poor college students. It shows that the degree of pressure it brings to students is not deep, but it does not mean that we can ignore this factor, nor does it mean that it will not become the main factor for some individuals to produce psychological crisis. Major and unexpected events are likely to bring a certain degree of pressure to students with poor psychological tolerance, so psychological health educators should pay special attention to these students.

### Exploratory and impedance factor analysis of statistical data

**Figure 4:**
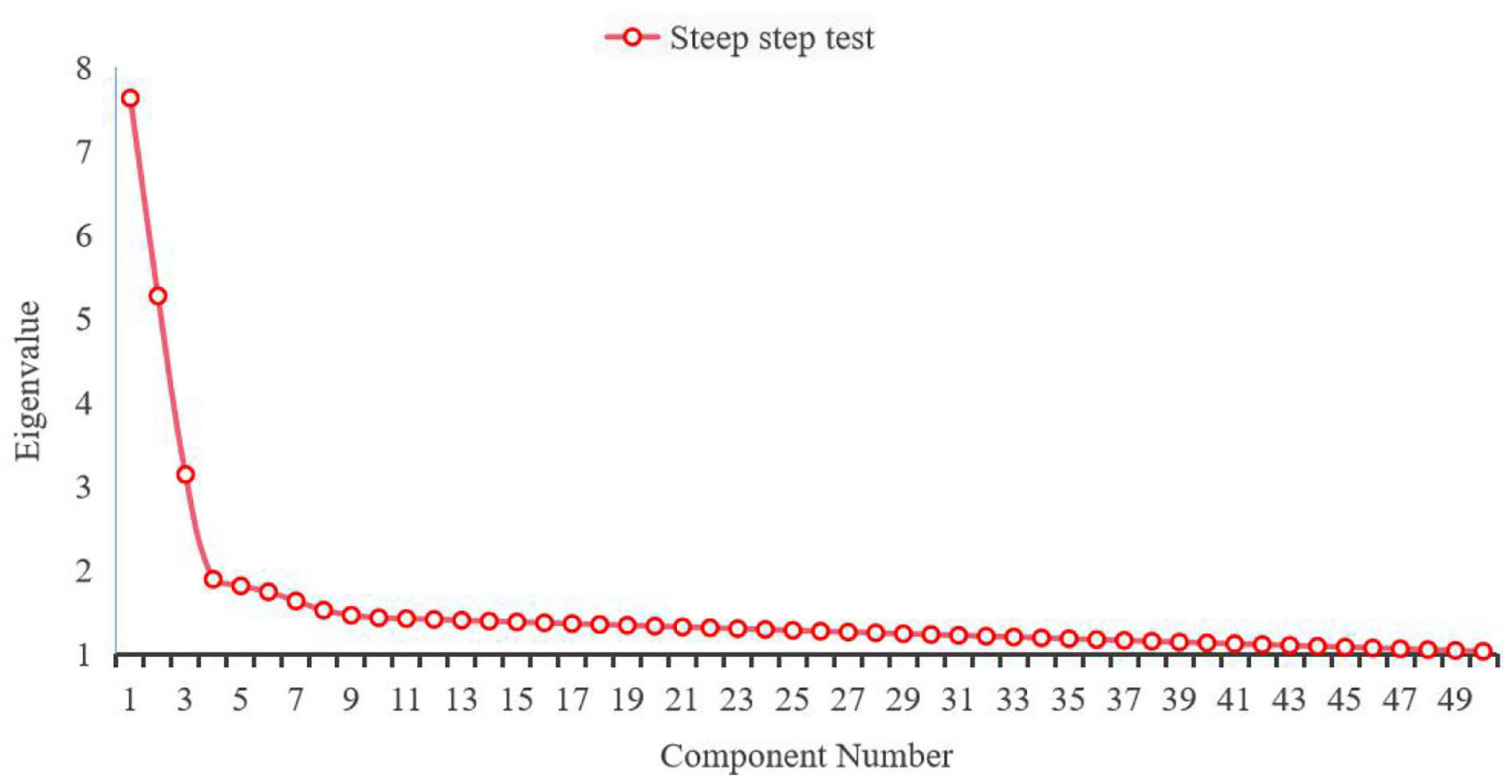
Gravel diagram of steep step inspection.

To test whether the survey data are suitable for factor analysis, this paper conducts sphericity test on the data, conducts exploratory factor analysis on all items of the questionnaire, and uses principal components and maximum orthogonal rotation method to extract factors. This was supplemented by the total explanation rate and steepness test to determine the number of factors. The steepness test of the characteristic graph advocated by the eigenvalues of the factors is shown in Figure 4.

The resistance of the educated is due to the inner quality factor of the educator. It mainly includes the reflection of students' attitudes toward the educator's character and charisma. The various dimensions of the questionnaire should have a moderate degree of correlation. If the correlation is too high, it means that there is overlap between the dimensions, and some dimensions are unnecessary. If the correlation is too low, it means that some completely different psychological qualities are being measured.

**Figure 5:**
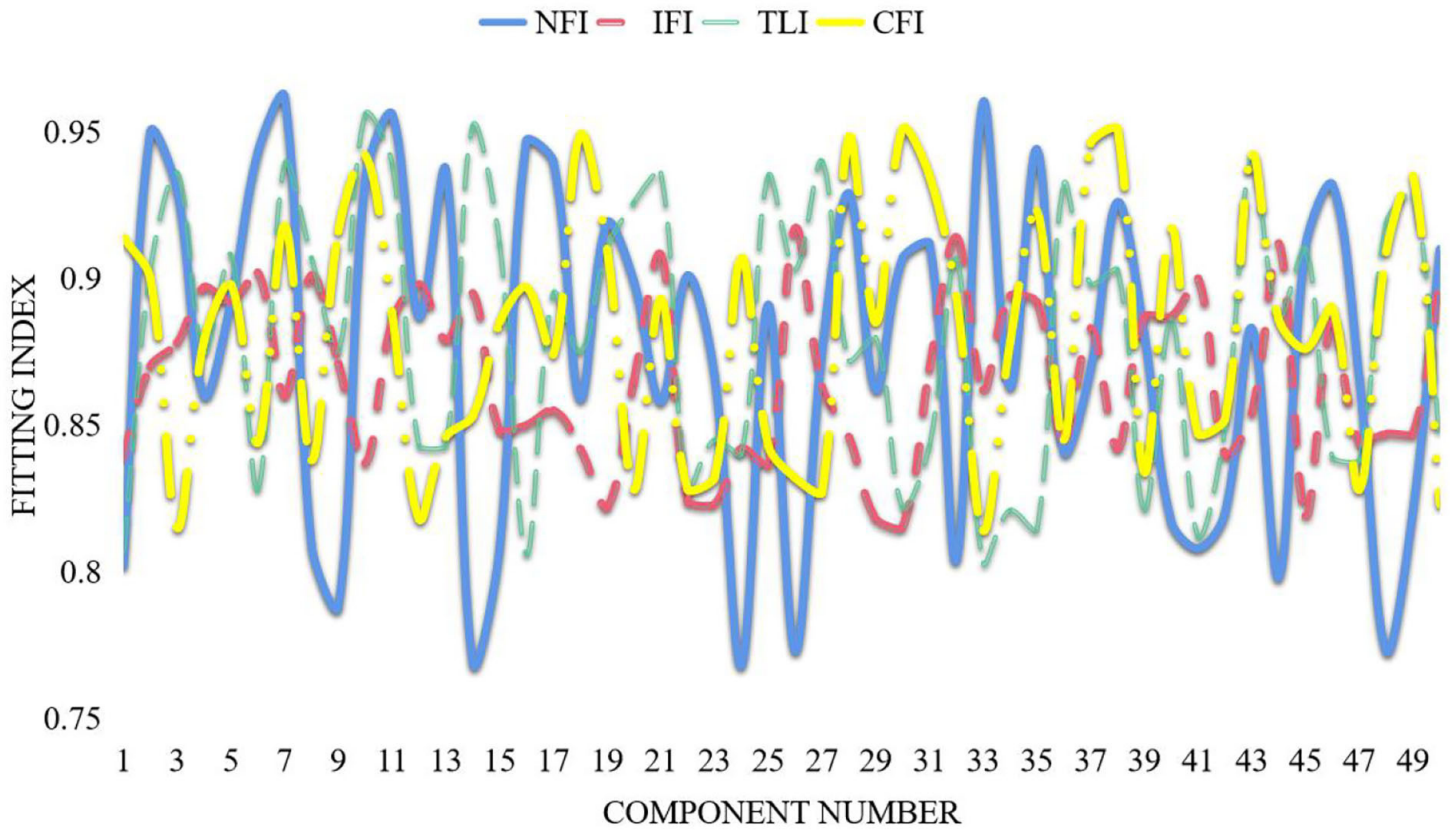
Fitting index of the model.

To examine the fitting degree between the structural model and the actual model, as well as the relationship between the project and each factor, this paper conducts a confirmatory factor analysis of the model. The factor structure model and standardization coefficient are shown in Figure 5.

It can be seen from Figure 5 that the correlation coefficient between the various factors fluctuates between 0.76 and 0.98, indicating that the factors are different but the same content is measured, and the correlation coefficients have reached an insignificant level. Psychological resistance is a clinical concept in the field of psychological counseling and therapy. The behaviorist school and the cognitive psychology school have different views. They believe that resistance is an individual's disobedience to its behavior modification. It is either due to the individual's doubts about counseling or to the individual's lack of environmental conditions for behavioral change.

**Figure 6:**
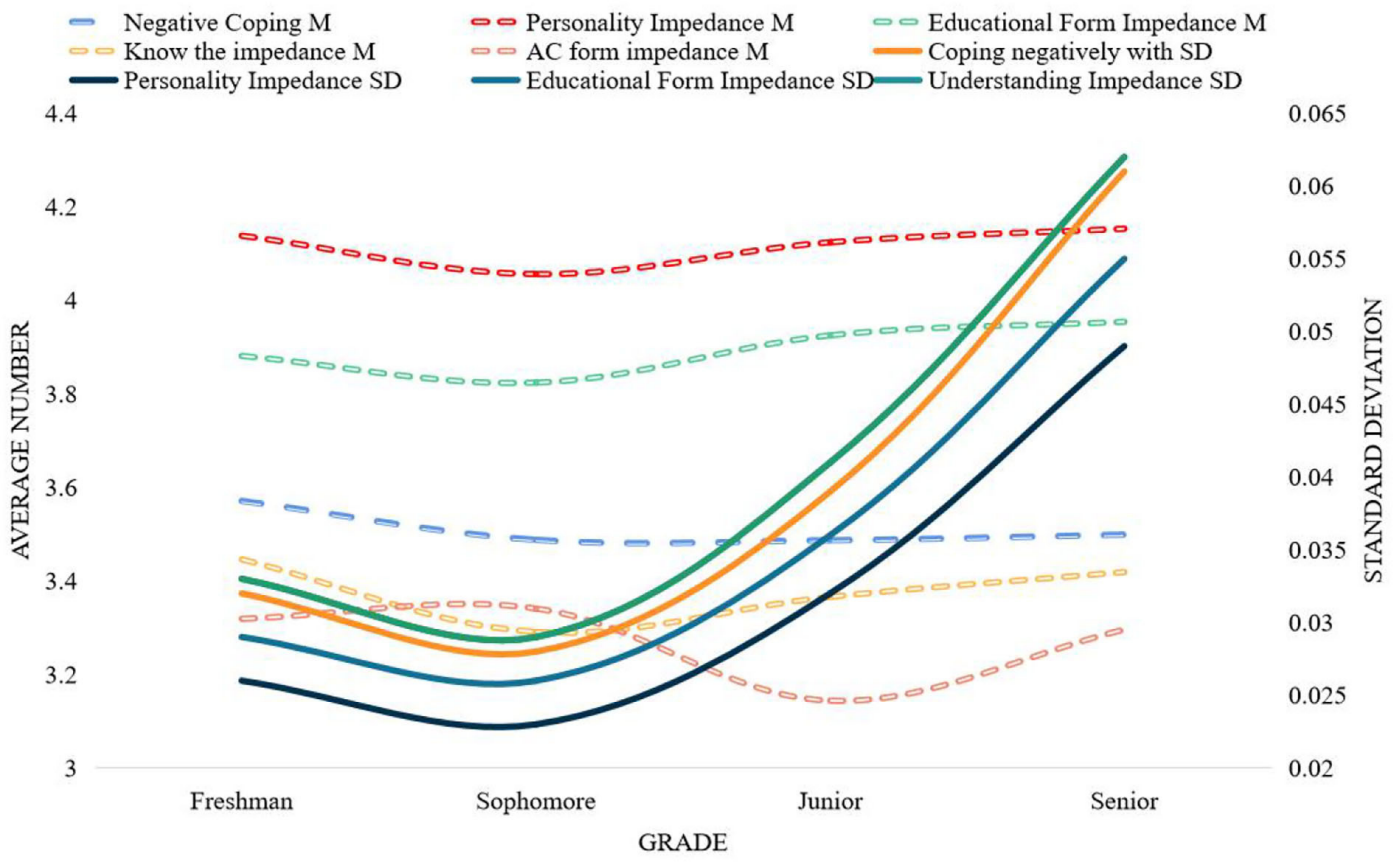
Mean and standard deviation of each factor score for grades.

The humanistic school is different from the previous two views. It believes that psychological resistance is the individual's resistance to self-exposure and emotional experience, and its purpose is to make the individual's self-knowledge and self-esteem not threatened. Ideological education is the medium of education. At the same level, it also includes all the elements of education. The general laws of education, such as the laws of adaptation and transcendence, the laws of two-way interaction, and the laws of internalization and externalization, are restricted by the basic laws, and the psychological state and the relationship between the educator and the educated in the process. The psychological interaction mechanism also has an important impact on the effect of education. In this regard, this paper believes that the psychological resistance in the process of ideological education is the student's influence on the ideological and political educator, educational content, educational form, educational process, and educational strategies in the process of ideological education. The implicit or explicit confrontational behavior that appears is the refusal of certain behaviors and cognitive changes to be made under the influence of ideological education, usually showing rejection behaviors and aversion to ideological education. This phenomenon is essentially the cognitive and psychological reaction of students to the process of ideological education and its various elements, reflecting the degree to which students receive ideological education. This paper analyzes the average score and standard deviation of the impedance of each factor in the scheduling data, as shown in Figure 6.

Figure 6 shows the differences of college students' psychoeducational impedance in different grades and the changing trend of each factor dimension. From a longitudinal point of view, the psychological education impedance of college students is, from high to low grades, personality impedance, educational form impedance, negative coping, cognitive impedance, and communication form impedance. From a horizontal perspective, the scores of the first-year students in the two dimensions of negative coping and cognitive resistance are higher than those of the other three grades; in the two dimensions of personality resistance and educational form resistance, the senior students have the highest scores, except for the communication form. In addition to impedance, the scores of sophomores in the other four dimensions are significantly lower than those of other grades; the scores of juniors in the dimension of communication form impedance are significantly lower than those of the other three grades.

The psychological resistance phenomenon of college students in the process of ideological education is reflected as the level of students' acceptance and internalization of the content of ideological education at the cognitive and psychological levels, and its manifestations are implicit and explicit. On the one hand, the resistance phenomenon of some students is not easy to detect, with strong concealment and deception. The psychological resistance phenomenon of students in the process of ideological education has a strong orientation, which is resistance to specific content in specific fields. For example, some students have resistance to beliefs and ideals in ideological education. They are extremely disgusted with beliefs and ideals due to the influence of factors such as family, economy, emotion, existing cognitive models, and bad emotional experiences in the process of their own growth, do not want to talk about, like unrestrained, free indulgence, and life pays attention to the principle of reality and the principle of happiness. In the process of ideological education, the psychological resistance of students is often based on the individual, but at the same time has a strong diffusion. Some students will influence each other due to their hobbies, similar personalities, and peer group relationships. In the process of ideological education, the resistance behavior of individual students will arouse the resonance or participation of other students and form small groups. Frontline ideological and political educators must clearly know the specific manifestations of the psychological resistance of college students in the process of ideological education, which is the key to improving the pertinence and effectiveness of countermeasures.

### Construction of structural equation model for psychological early warning with interactive effects

**Figure 7:**
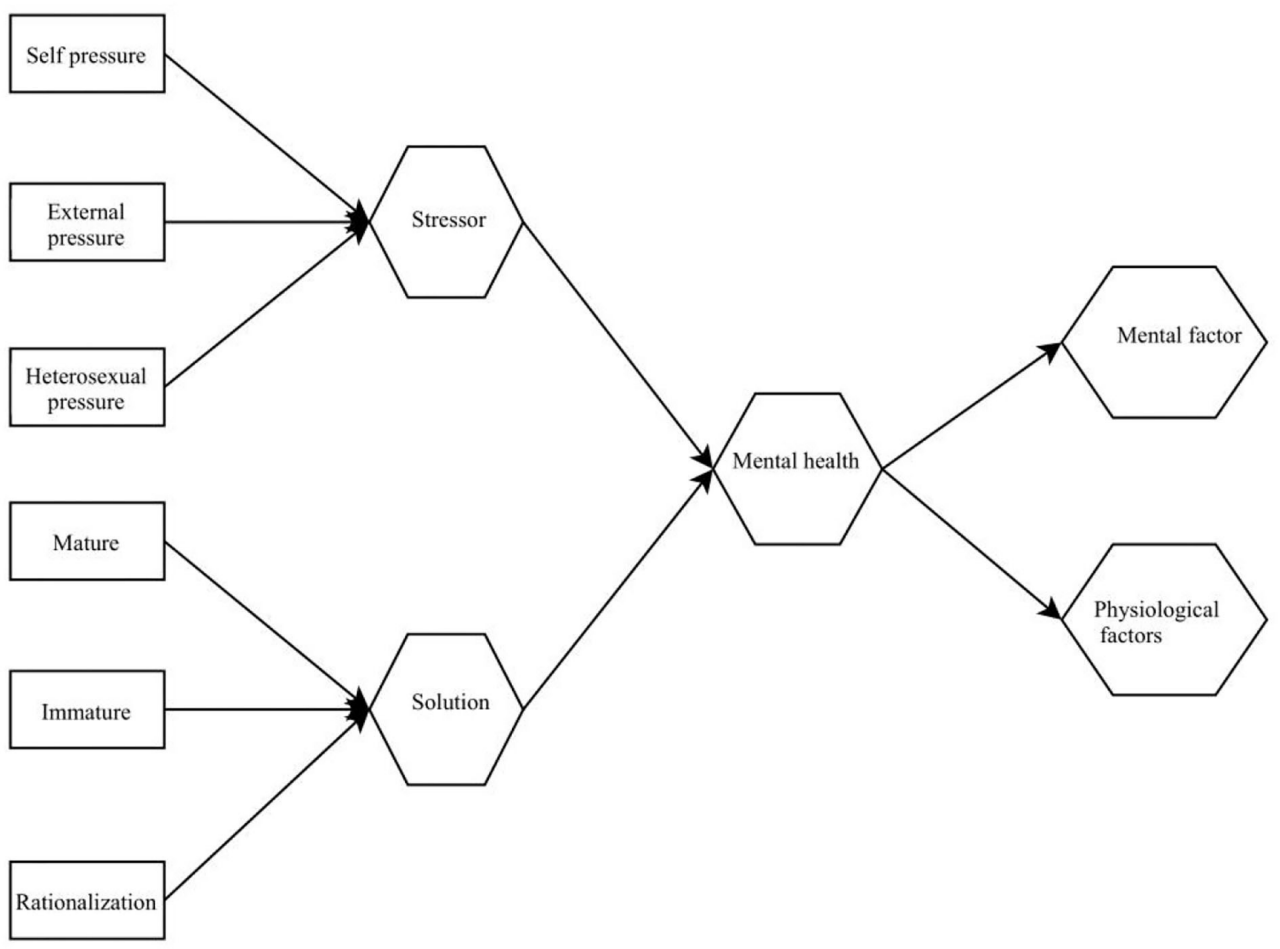
Basic pathways of stressors and coping styles.

This paper takes psychological health as the endogenous latent variable, describes the observed variables as mental factors and physiological factors, takes the stressor as the first exogenous latent variable, and describes its observed variables as self-pressure, external pressure, and heterosexual pressure as coping methods. For the second exogenous latent variable, the observed variables that characterize it are mature coping (x), immature coping, and rational coping. According to the basic path structure of stressors and coping styles in Figure 1, a structural equation model is established as shown in Figure 7 shown.

**Figure 8:**
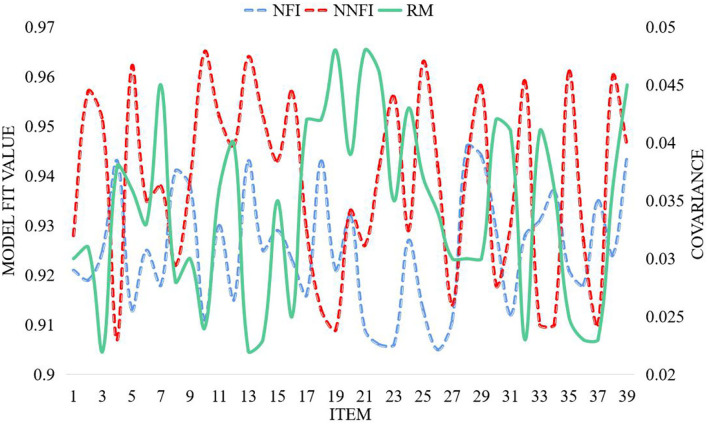
Model fit test.

According to the path analysis of stressors, coping styles, and psychological health, the model fitting test of the direct influence of stressors on psychological health was constructed, as shown in Figure 8.

It can be seen from Figure 8 that the goodness-of-fit index NFI is >0.9, NNFI is >0.9, and RM is <0.05, indicating that the model fits well and the model is acceptable. By comparison, it is not difficult to find that stressors have a greater impact on psychological health. Among the factors affecting coping style, immature coping style ranks first, followed by mature coping style and rationalized coping style. It shows that compared with the mature and rational coping styles, the immature coping style can affect psychological health more. A significant positive correlation between stressors and coping styles can be seen from the structural pathway diagram. The factor that had the greatest impact on psychological health was the stressor, followed by the interaction term between the stressor and coping style, and finally the coping style. The path coefficient between coping style and psychological health in this model is negative, because the addition of the interaction term makes the weight of each factor affecting coping style change greatly. Mature coping is ranked first, followed by rationalization, and finally immature coping. The mature coping style with a larger proportion is significantly negatively correlated with the total score of psychological health, so the obtained coefficient is negative number. From the structural path diagram, it can be found that stressors, coping styles, and interaction terms are significantly positively correlated in pairs. The influence of stressors on psychological health has slightly decreased, but it is still in a significant range. The influence of coping style on psychological health has been greatly weakened, from the previous significant level to the current insignificant level. Observing the coefficients of the main effect and the interaction effect, it is not difficult to find that the influence of the interaction term on psychological health is not equal to the sum of the influence of the two main effects, but less than the superposition of the two. Early detection of potential or actual crisis factors, effective measures to reduce the occurrence of crises, and prevention are the purpose of psychological crisis early warning. After the psychological crisis early warning mechanism is established, the psychological crisis assessment mechanism should be activated. The crisis intervention stereotype assessment tool can be divided into the following levels. The first level emotional state is stable, and the emotional expression of daily activities is appropriate. The second level of emotional response to the environment is appropriate, and there is only a short-term negative emotional expression to the changes of the environment. The caller is completely able to control his emotions. The third level of emotional response to the environment is appropriate, and the caller can realize the need for self-control. The fourth level emotional response is out of touch with the environment. They often show negative emotions and have strong emotional fluctuations about environmental changes. The fifth level of negative emotional experience significantly exceeds the impact of the environment. Emotion is obviously out of harmony with the environment. The caller is aware of negative emotions, but cannot control them. Level 6 is completely out of control or extremely sad. Based on the information of the high-risk groups of psychological crisis screened out by the system, the organization should conduct psychological assessment on these college students, taking into account the students' life, study, and other aspects, determine the alert object.

## Conclusion

Based on the results of early warning evaluation, this paper takes different intervention measures according to the degree of students' psychological crisis. The study found that there was a significant correlation between stressors and mental health. It is one of the many factors that affect mental health, and it is also the main reason to induce college students' psychological crisis. Mature coping styles are significantly positively correlated with mental health, while immature coping styles are significantly negatively correlated with mental health. Mature coping styles play a positive role in promoting mental health. Mental health has a negative impact, which is significantly greater than the promotion of mature coping styles. Through the fitting test of the model, it is found that the goodness-of-fit index NFI is >0.9, NNFI is >0.9, and RM is <0.05, indicating that the model fits well and the model is acceptable. For students with a low degree of psychological crisis, routine mental health care is needed, that is, to carry out mental health education, psychological counseling, alleviate personal learning and life pressure, and maintain a positive attitude. For students with a warning level of crisis, peer guidance and more social support are needed. In terms of research methods, this paper only conducted a test, that is, to investigate the subjects' stress tolerance and coping style at a certain point in time, and to study the relationship between the variables. Individual students' stress tolerance and coping styles may change over time. In this paper, students should be tested regularly, which is more conducive to the timely warning of psychological crisis. This paper lacks the formulation of emergency plan. In view of the various possibilities of crisis events, we choose the appropriate emergency psychological plan to effectively deal with the crisis. We should plan, assign, and organize the crisis management team of colleges and universities in advance to avoid the rush and disorder of temporary formation when the crisis occurs.

## Data availability statement

The original contributions presented in the study are included in the article/supplementary material, further inquiries can be directed to the corresponding author.

## Ethics statement

Ethical review and approval was not required for the study on human participants in accordance with the local legislation and institutional requirements. Written informed consent from the patients/participants or patients/participants legal guardian/next of kin was not required to participate in this study in accordance with the national legislation and the institutional requirements.

## Author contributions

CL: writing and editing. KL: data collection. Both authors contributed to the article and approved the submitted version.

## Conflict of interest

The authors declare that the research was conducted in the absence of any commercial or financial relationships that could be construed as a potential conflict of interest.

## Publisher's note

All claims expressed in this article are solely those of the authors and do not necessarily represent those of their affiliated organizations, or those of the publisher, the editors and the reviewers. Any product that may be evaluated in this article, or claim that may be made by its manufacturer, is not guaranteed or endorsed by the publisher.
